# Interactions of spontaneous abortion with *FTO* gene and dietary carotenoids; a case–control study

**DOI:** 10.1017/jns.2024.55

**Published:** 2024-11-27

**Authors:** Arezoo Amjadi, Khadijeh Abbasi Mobarakeh, Saeid Doaei, Masoumeh Dorosti, Sheyda Nami, Seyed Reza Mirshafaei, Masoomeh Alsadat Mirshafaei, Masoomeh Ataei Kachooei, Ali Shamsi-Goushki, Zahra Saeedirad, Ghasem Azizi Tabesh, Sara Khoshdooz, Morteza Abdollahi, Soheila Shekari, Maryam Gholamalizadeh

**Affiliations:** 1 Department of Nutrition, School of Nutritional Sciences and Food Technology, Kermanshah University of Medical Sciences, Kermanshah, Iran; 2 Department of Community Nutrition, Nutrition and Food Security Research Center, School of Nutrition and Food Science, Isfahan University of Medical Sciences, Isfahan, Iran; 3 Department of Community Nutrition, National Nutrition and Food Technology Research Institute, Faculty of Nutrition Sciences and Food Technology, Shahid Beheshti University of Medical Sciences, Tehran, Iran; 4 Reproductive Health Research Center, Department of Obstetrics and Gynecology, School of Medicine, Al-Zahra Hospital, Guilan University of Medical Sciences, Rasht, Iran; 5 Student Research Committee, Department of Nutrition, Faculty of Medicine, Urmia University of Medical Sciences, Urmia, Iran; 6 Department of Clinical Biochemistry, Iran University of Medical Sciences, Tehran, Iran; 7 Department of Applied Mathematics, Roudsar and Amlash branch, Islamic Azad University, Roudsar, Iran; 8 Islamic Azad University, Tonekabon Mazandaran Branch, Tonekabon, Iran; 9 Faculty of Medicine, Shahrekord, University of Medical Sciences, Shahrekord, Iran; 10 Department of Nutrition, School of Medicine, Mashhad University of Medical Sciences, Mashhad, Iran; 11 Department of Medical Genetics, School of Medicine, Genomic Research Center, Shahid Beheshti University of Medical Sciences, Tehran, Iran; 12 Faculty of Medicine, Guilan University of Medical Sciences, Rasht, Iran; 13 Social Determinants of Health Research Center, and National Nutrition and Food Technology Research Institute, Faculty of Nutrition Sciences and Food Technology, Shahid Beheshti University of Medical Sciences, Tehran, Iran; 14 Department of Nutrition, Science and Research Branch, Islamic Azad University, Tehran, Iran; 15 Cancer Research Center, Shahid Beheshti University of Medical Sciences, Tehran, Iran

**Keywords:** Antioxidant, Carotenoid, *FTO* gene, Oxidative stress, Spontaneous abortion, SA, Spontaneous abortion, WHO, World Health Organization, FTO, fat mass and obesity-associated, ARMS-PCR, amplification refractory mutation system-polymerase chain, FFQ, food frequency questionnaire

## Abstract

Spontaneous abortion (SA) is considered one of the most prevalent adverse outcomes of pregnancy. SA may occur due to genetic susceptibility and various maternal factors such as nutritional status. The aim of this study was to assess how dietary carotenoids and the *FTO* gene are related to SA. This case–control study included 192 women with a history of SA as the case group and 347 healthy women without history of SA as the control group. To evaluate carotenoid intake, a valid 168-item food frequency questionnaire (FFQ) was used. The *FTO* gene was genotyped for the presence of the *rs9939609* polymorphism using the tetra-primer amplification refractory mutation system-polymerase chain (ARMS-PCR). The results indicated a significant negative association between dietary intake of β-cryptoxanthin and SA in carriers of the TT genotype of the *FTO rs9939609* polymorphism after adjustment for age, BMI, physical activity, smoking, alcohol drinking, and calorie intake (β = −0.28, P = 0.02). No association was found between SA with dietary intake of beta-carotene, alpha-carotene, lutein, and lycopene among carriers of different *FTO* genotypes. The *FTO* genotype may have an effect on the association between SA and carotenoid intake. Dietary intake of β-cryptoxanthin may act as a protective factor against SA only in carriers of the TT genotype of the *FTO rs9939609* polymorphism.

## Introduction

Spontaneous abortion (SA) is one of the most common issues in early pregnancy, usually caused by chromosomal abnormalities and hormonal and physiologic problems^(1)^. According to the World Health Organization (WHO), SA can be defined as the loss of a fetus weighing equal to or less than 500 g, typically occurring in the first 20 weeks of gestation^(2,3)^. Moreover, abortion can lead to complications such as hemorrhage requiring blood transfusion, and infection in the upper genital tract that leads to endometritis, oophoritis, para metritis, and salpingitis^(4)^. The most known risk factors for SA are genetic disorders, chromosomal abnormalities, infectious diseases, age of the mother, and abortion history^(1)^. Overall, 55.7 million abortions are estimated to take place each year in the world^(1)^. However, in Iran, accurate data on abortion is not available^(5)^, the prevalence of abortion in Iran in 2012 was estimated between 70.5 and 116.9 per 1000 pregnancies^(5)^.

The fat mass and obesity-associated (*FTO*) gene was initially discovered through a genome-wide association study and has been identified as an independent genetic risk factor for obesity^(5)^. The *FTO* gene is believed to be involved in the demethylation of nucleic acids, a process that can affect gene expression. Additionally, correct fetal development relies on various factors including DNA methylation-based programming. Considering the role of the *FTO* gene in demethylation processes, it is plausible to hypothesize that variations in this gene could potentially impact fetal development and contribute to a predisposition to SA^(6)^. In this direction, the *FTO rs9939609* single nucleotide polymorphism was identified as a risk factor for SA in a cohort study of 202 Sinhalese women with a history of SA and 202 normal control women^(7)^. Previous studies have suggested that decreased *FTO* -mediated demethylation in trophoblasts may be a potential underlying cause of SA^(8)^.

On the other hand, a possible link has been suggested between dietary factors and reproductive risks^(9)^. A dysfunctional maternal-fetal interface can lead to oxidative stress in the placenta, which in turn can result in the loss of placental synthetic trophoblast cells. This process contributes to the development of abortion^(10)^. The lower levels of dietary antioxidants may aggravate pro-oxidative injury in endothelial cells, cause changes in thromboxane-prostacyclin balance, and may contribute to preeclampsia and SA^(11)^. Dietary carotenoids have many biological properties including antioxidant, anti-inflammatory, and immunomodulatory effects^(12–14)^, and therefore, they may have a protective effect against abortion. Some studies indicated that carotenoids play an important role in pregnancy outcomes and in the prevention of many pathologies of pregnancy that are brought about by increased oxidative stress^(15–17)^. Furthermore, some types of carotenoids are known as vitamin A precursor and the World Health Organization (WHO) approximates that approximately 19 million pregnant women in low-income countries suffer from vitamin A deficiency^(18)^. The findings from the Norwegian Mother and Child Cohort Study (MoBa) demonstrate that the plasma levels of carotenoids in pregnant women are directly associated with their intakes of fruits and vegetables^(19)^. Therefore, during pregnancy and lactation a diet rich in vegetables and fruits as a source of carotenoids may be beneficial against vitamin A deficiency^(12)^.

Moreover, the effect of dietary carotenoids on human health was reported to be influenced by FTO genotype^(20,21)^. However, there are no studies on the relationship between carotenoids and miscarriage in individuals with different *FTO* polymorphisms. The impact of *FTO* gene variations on SA risk and how it interacts with carotenoids would require targeted investigations. So, the present study was designed to evaluate the interactions between SA, the dietary intake of carotenoids, and the genotype of *FTO* gene.

## Methods

This study was initially conducted on 600 adult women, including 200 women with a history of SA and 400 women without a history of abortion in Tehran, Iran. Participants were randomly selected from among those referred for general check-ups to the Shohadaye Tajrish hospital, Tehran, Iran. The inclusion criteria for the case group were a history of at least one SA before the 20th week of pregnancy and an age between 20 and 40 years. The inclusion criteria for the control group were no history of SA and an age between 20 and 40 years. Individuals who were taking carotenoid supplements (n = 2) and did not have the desire to continue participating in the study (n = 27) or were unable to provide the necessary information (n = 32) were excluded from the study. Finally, 539 women (192 cases and 347 controls) were included. At the beginning of the study, the purpose and method of the study were explained to all participants, and the written consent was obtained.

Data related to age, education level, smoking, alcohol consumption, and history of reproductive system diseases, diabetes, hypertension, abortion, and pregnancy were collected using a general questionnaire through face-to-face interviews. The height of individuals was measured using a stadiometer with an accuracy of 0.5 cm, and weight was measured using a digital scale with an accuracy of 0.5 kg.

### Investigation of the *FTO* gene genotype

To evaluate the *FTO* gene genotype for the presence of the *rs9939609* polymorphism, 5 cc of blood was collected from all participants. Then, blood cells were separated using the centrifugation method, and DNA was extracted using a standard kit. DNA samples were amplified using the polymerase chain reaction (PCR) method and master mix polymerase (Cat. No. A180301; Amplicon Denmark). The tetra-primer amplification refractory mutation system-PCR (TETRA ARMS-PCR) method was used to identify the *rs9939609* polymorphism of the *FTO* gene.

### Carotenoid intake

In order to evaluate the amount of dietary carotenoid intake, a semi-quantitative, valid 168-item food frequency questionnaire (FFQ) was used^(22)^. The collected information was converted to daily nutrient intake using Nutritionist IV software (First Databank, San Bruno, CA, USA), and the intake of various types of carotenoids including beta carotene, alpha carotene, lutein, beta cryptoxanthin, and lycopene was estimated.

### Statistical analysis

Normal distribution of data was confirmed using the Kolmogorov–Smirnov test. To compare social-demographic indices and food intake, the chi-square and independent t-test methods were used for qualitative and quantitative variables, respectively. Linear regression was used to investigate the relationship between the amount of carotenoid intake and the number of SAs after adjustment for other carotenoids (Model 1), further adjustment for age (Model 2), further adjustment for BMI, physical activity, smoking, and alcohol drinking (Model 3), and further adjustment for calorie intake (Model 4). Additionally, a linear regression analysis was performed to investigate the relationship between SA and carotenoid intake, separately for individuals with AA/AT and TT genotypes of the *FTO* gene based on the dominant genetic model. The variance inflation factor (VIF) was used for the regression model to investigate the potential of collinearity among independent variables. All variables had a VIF <2 and thus multicollinearity was not significant. All analyses were performed using SPSS software version 27, and a significance level of P<0.05 was considered.

## Results

The general characteristics of the participants are presented in Table 1. The cases had higher alcohol consumption and a lower history of diabetics (both *P*<0.05). No significant difference was found in terms of age, weight, height, BMI, first menstruation age, right diastolic blood pressure (DBP), right systolic blood pressure (SBP), white blood cells (WBC), red blood cells (RBC), hemoglobin (HGB), hematocrit (HCT), mean corpuscular volume (MCV), mean corpuscular hemoglobin (MCH), Mean corpuscular hemoglobin concentration (MCHC), platelet, lymphocyte, monocyte, blood urea nitrogen (BUN), creatinine (Cr), triglycerides (TG), cholesterol, glutamic-oxaloacetic transaminase (SGOT), aspartate aminotransferase (AST), alkaline phosphatase (ALP), High-density lipoprotein cholesterol (HDL-C), low-density lipoprotein-cholesterol (LDL-C), PCR Results on FTO genotypes, using Tobacco, and having hypertension.

**Table 1.** General characteristics of the participants

	Cases (n = 192)	Controls (n = 347)	P
Age (year)	24.84 ± 101.60	25.60 ± 12.86	0.07
Height (cm)	156.87 ± 6.19	156.80 ± 5.68	0.88
Weight (kg)	71.89 ± 10.51	70.35 ± 10.29	0.10
BMI (kg/m^2^)	29.16 ± 4.01	28.59 ± 3.96	0.11
Mens First Age (year)	13.39 ± 1.51	13.27 ± 1.62	0.50
Right DBP (mmHg)	70.52 ± 9.33	70.34 ± 8.80	0.85
Right SBP (mmHg)	109.29 ± 14.03	108.75 ± 14.19	0.73
WBC (K/µL)	6.33 ± 1.52	6.15 ± 1.42	0.28
RBC (M/µL)	4.83 ± 0.40	4.85 ± 0.38	0.59
HGB (gr/dl)	13.35 ± 1.09	13.33 ± 0.96	0.28
HCT (%)	40.53 ± 2.88	40.55 ± .2.68	0.94
MCV (fL)	84.16 ± 6.04	83.75 ± 5.53	0.54
MCH (pg)	27.72 ± 2.42	27.55 ± 2.18	0.51
MCHC (gr)	32.91 ± 0.79	27.55 ± 2.18	0.62
Platelets (K/µL)	316.11 ± 66.48	308.20 ± 68.68	0.29
Lymphocyte (10^6^/L)	40.97 ± 8.07	41.23 ± 8.92	0.77
Monocyte (10^6^/L)	3.25 ± 1.03	3.28 ± 1.06	0.81
BUN (mg/dl)	12.50 ± 3.17	12.62 ± 3.51	0.74
Creatinine (mg/ml)	0.97 ± 0.11	0.96 ± 0.11	0.72
TG (mg/dl)	133.35 ± 79.82	121.55 ± 69.11	0.16
Cholesterol (mg/dl)	195.66 ± 37.24	194.52 ± 39.65	0.79
SGOT (IU/L)	19.04 ± 5.51	18.49 ± 4.87	0.35
SGPT (IU/L)	18.87 ± 7.62	17.70 ± 9.04	0.19
ALP (IU/L)	233.73 ± 76.95	220.18 ± 66.45	0.09
HDLC (mg/dl)	54.39 ± 10.78	55.76 ± 11.44	0.26
LDL (mg/dl)	114.59 ± 32.52	114.45 ± 33.39	0.97
PCR result (%)			0.33
TT	94 (36.96)	40 (28.6)	
AA	22 (8.6)	14 (10.0)	
AT	138 (54.1)	86 (61.4)	
Use Alcohol (yes, n, %)	17 (8.9)	28 (8)	0.001
Tobacco (yes, n, %)	20 (5.8)	8 (4.2)	0.27
Has Diabetes (yes)	81 (42.2)	60 (17.29)	0.03
Has Hypertension (yes)	49 (25.6)	94 (27.1)	0.39

Right SBP: Right Systolic Blood Pressure, Right DBP: Right Diastolic Blood Pressure, WBC: white blood cell, RBC: red blood cell, BUN: Blood Urea Nitrogen, TG: Triglyceride, HDL-c: high-density lipoprotein cholesterol, LDL-c: low-density lipoprotein cholesterol, SGOT: Serum Glutamic Oxaloacetic Transaminase, SGPT: Serum Glutamic pyruvic Transaminase, ALP: Alkaline phosphatase.

The dietary intake of participants is shown in Table 2. There was no significant difference in dietary intake of micronutrients and macronutrients between the cases and controls. Also, as shown in Table 3, no significant difference was observed in dietary intakes in carriers of different *FTO* genotypes. The linear regression of the association between SA and dietary carotenoids is indicated in Table 4. There was no significant association between abortion and dietary carotenoids.

**Table 2.** Dietary intakes of the participants

	Cases	Controls	P
Calorie (kcal/day)	2534.83 ± 437.91	2552.82 ± 674.69	0.74
Protein (g/day)	84.71 ± 21.34	83.79 ± 30.31	0.71
Carbohydrates (g/day)	359.44 ± 73.84	363.17 ± 99.81	0.66
Fats (g/day)	92.13 ± 21.74	94.16 ± 31.69	0.44
Cholesterol (g/day)	268.86 ± 157.41	258.201 ± 136.07	0.49
SFA (g/day)	28.92 ± 9.72	28.82 ± 13.78	0.93
MUFA (g/day)	34.24 ± 14.10	33.48 ± 15.92	0.62
PUFA (g/day)	18.66 ± 8.01	19.47 ± 8.88	0.34
Omega 3_Fatty acid (g/day)	1.23 ± 0.69	1.22 ± 0.80	0.93
Omega 6_Fatty acid (g/day)	4.83 ± 5.01	5.22 ± 5.52	0.53
Na (mg/day)	6995.05 ± 4655.22	6791.88 ± 4227.66	0.68
K (mg/day)	5280.69 ± 3614.65	5651.81 ± 4193.47	0.37
Vitamin A (mcg/day)	677.55 ± 386.77	709.45 ± 986.93	0.64
Beta carotene (mcg/day)	5091.39 ± 3758.45	5424.87 ± 11608.65	0.67
Alpha carotene (mcg/day)	39292.21 ± 56689.17	49050.87 ± 177068.11	0.47
Lutein (mcg/day)	2000.27 ± 1369.08	2182.12 ± 4007.53	0.51
Beta cryptoxanthin (mcg/day)	428.31 ± 358.30	450.50 ± 343.89	0.57
Lycopene (mcg/day)	8799.23 ± 7008.36	9210.39 ± 8372.30	0.63
Vitamin C (mg/day)	199.75 ± 169.10	198.86 ± 162.17	0.96
Ca (mg/day)	1194.42 ± 428.43	1291.53 ± 682.78	0.07
Fe (mg/day)	18.15 ± 5.26	17.94 ± 6.97	0.73
Vitamin D (mcg/day)	1.97 ± 1.31	1.83 ± 1.39	0.32
Vitamin E (mg/day)	16.62 ± 8.26	18.03 ± 9.69	0.12
Alpha tocopherol (mg/day)	11.86 ± 5.27	12.46 ± 7.70	0.34
Thiamin (mg/day)	2.00 ± 0.51	2.04 ± 0.77	0.52
Riboflavin (mg/day)	2.18 ± 0.61	2.29 ± 1.03	0.20
Niacin (mg/day)	21.38 ± 7.25	21.18 ± 8.44	0.80
Vitamin B6 (mg/day)	1.89 ± 0.63	1.92 ± 0.74	0.69
Folate (mcg/day)	704.14 ± 208.22	666.17 ± 274.56	0.11
Vitamin B12 (mg/day)	4.42 ± 2.32	4.56 ± 3.18	0.62
Biotin (mcg/day)	31.46 ± 12.02	31.39 ± 13.53	0.95
Pantothenic (mg/day)	5.57 ± 1.63	5.61 ± ± 2.02	0.80
Vitamin K (mg/day)	5280.69 ± 3614.65	5651.81 ± 4193.47	0.50
Phosphorus (mg/day)	143402.07 ± 195401.66	146208.575 ± 267926.76	0.91
Mg (mg/day)	410.81 ± 117.21	419.02 ± 204.19	0.60
Zinc (mg/day)	11.83 ± 4.34	12.04 ± 5.19	0.65
Cu (mg/day)	1.91 ± 0.62	1.84 ± 0.83	0.33
Manganese (mg/day)	5.67 ± 1.80	5.67 ± 2.59	0.99
Selenium (mg/day)	96.22 ± 32.72	96.10 ± 38.43	0.97
Fluorine (mg/day)	2707.35 ± 1890.964	2553.71 ± 1993.25	0.46
Chrome (mcg/day)	0.06 ± 0.07	0.07 ± 0.08	0.48
Total fibre (g/day)	31.76 ± 13.54	31.01 ± 17.78	0.62
Soluble fibre (g/day)	1.74 ± 1.41	1.77 ± 1.86	0.87
Insoluble fibre (g/day)	472.15 ± 611.76	505.34 ± 32.87	0.72
Crude fibre (g/day)	35.49 ± 36.03	30.20 ± 28.36	0.18
Sugar (g/day)	144.65 ± 52.98	150.99 ± 58.61	0.26
Glucose (g/day)	21.08 ± 12.77	20.92 ± 10.92	0.90
Galactose (g/day)	4.51 ± 3.02	4.84 ± 5.75	0.46
Fructose (g/day)	24.99 ± 14.26	23.91 ± 12.43	0.45
Sucrose (g/day)	55.45 ± 39.25	56.57 ± 37.64	0.78
Lactose (g/day)	16.16 ± 9.25	16.71 ± 16.15	0.66
Maltose (g/day)	2.11 ± 1.12	2.18 ± 1.42	0.59
Caffeine (mg/day)	115.15 ± 110.34	120.34 ± 123.75	0.80

**Table 3.** Dietary intake of the participants considering *FTO* rs9939609 genotypes

	TT			AA			AT		
	Cases	Controls	P	Cases	Controls	P	Cases	Controls	P
Calorie (kcal/d)	2551.98 ± 406.06	2564.6 ± 592.36	0.89	2422.01 ± 286.18	2670.9 ± 821.83	0.24	2543.77 ± 451.05	2546.13 ± 701.83	0.97
Protein (g/day)	83.78 ± 17.17	84.17 ± 25.52	0.92	76.26 ± 14.58	88.87 ± 48.22	0.31	83.19 ± 19.53	83.85 ± 33.23	0.86
Carbohydrates (g/day)	361.24 ± 69.30	364.95 ± 100.38	0.82	344.24 ± 63.79	379.29 ± 122.09	0.31	363.18 ± 61.93	362.45 ± 89.15	0.95
Fats (g/day)	93.68 ± 16.39	95.28 ± 29.49	0.71	88.01 ± 11.89	97.49 ± 26.64	0.19	92.82 ± 26.98	93.10 ± 33.55	0.95
Cholesterol (g/day)	270.61 ± 132.03	281.23 ± 140.58	0.71	274.34 ± 159.4	213.69 ± 124.73	0.28	281.67 ± 172.78	258.01 ± 139.61	0.34
SFA (g/day)	29.95 ± 8.94	28.21 ± 11.42	0.38	23.68 ± 7.86	28.02 ± 10.26	0.19	29.35 ± 10.54	29.24 ± 16.72	0.96
MUFA (g/day)	32.91 ± 10.84	34.42 ± 13.75	0.53	32.97 ± 12.73	34.28 ± 14.32	0.79	35.64 ± 16.08	32.29 ± 17.32	0.17
PUFA (g/day)	20.0 ± 8.46	20.22 ± 8.28	0.89	21.51 ± 6.73	23.25 ± 12.95	0.62	18.36 ± 8.29	18.61 ± 7.58	0.84
Omega 3_Fatty acid (g/day)	1.29 ± 0.80	1.28 ± 0.75	0.95	1.17 ± 0.67	1.43 ± 1.31	0.49	1.23 ± 0.65	1.18 ± 0.69	0.68
Omega 6_Fatty acid (g/day)	6.38 ± 5.32	6.77 ± 6.07	0.77	3.76 ± 4.88	5.98 ± 9.52	0.49	5.08 ± 5.30	4.83 ± 4.71	0.78
Na (mg/day)	7451.83 ± 5639.4	6798.97 ± 4018.6	0.56	5880.34 ± 4366.75	8549.26 ± 6321.8	0.23	7213.84 ± 4378.88	6825.79 ± 4118.76	0.57
K (mg/day)	6303.04 ± 4597.5	5624.0 ± 3958.01	0.49	2822.82 ± 3747.17	5527.44 ± 3326.45	0.06	5291.4 ± 3154.26	6152.32 ± 5005.19	0.17
Vitamin A (mcg/day)	802.63 ± 455.13	840.60 ± 1735.96	0.85	635.54 ± 366.86	625.72 ± 317.76	0.93	648.79 ± 366.02	648.96 ± 354.16	0.99
Beta_carotene (mcg/day)	5984.1 ± 5192.4	7107.9 ± 21372	0.67	5575.82 ± 2902.84	4673.04 ± 2883.31	0.45	4623.93 ± 3358.72	4866.0 ± 3122.69	0.63
Alpha_carotene (mcg/day)	43486.3 ± 76153.8	53845.5 ± 319310.4	0.45	30990.54 ± 38927.14	44311.95 ± 69706.86	0.59	41891.06 ± 61864.92	45621.95 ± 58699.81	0.73
Lutein (mcg/day)	2131.4 ± 1509.3	2708.0 ± 7363.0	0.53	1915.25 ± 1276.9	2332.82 ± 1290.16	0.41	2014.06 ± 1388.69	1991.51 ± 1245.33	0.91
Beta cryptoxanthin (mcg/day)	343.23 ± 336.83	569.34 ± 427.7	0.08	338.98 ± 283.11	424.88 ± 293.9	0.48	410.32 ± 347.03	432.94 ± 325.66	0.68
Lycopene (mcg/day)	9607.28 ± 7471.31	10602.0 ± 10217.9	0.61	4971.46 ± 4947.33	7084.72 ± 6172.51	0.42	8491.86 ± 6601.08	9106.24 ± 7842.41	0.61
Vitamin C (mg/day)	220.40 ± 203.39	218.29 ± 222.0	0.96	125.04 ± 72.97	183.93 ± 154.63	0.22	196.37 ± 164.79	200.09 ± 134.91	0.87
Ca (mg/day)	1257.68 ± 395.18	1236.39 ± 569.80	0.82	1219.62 ± 420.85	1371.68 ± 579.69	0.40	1168.13 ± 386.74	1337.4 ± 850.26	0.06
Fe (mg/day)	18.96 ± 5.40	18.88 ± 9.12	0.95	18.82 ± 5.05	18.78 ± 8.07	0.98	17.62 ± 4.50	17.43 ± 5.27	0.79
Vitamin D (mg/day)	2.06 ± 1.38	1.95 ± 1.46	0.71	2.36 ± 1.77	1.81 ± 1.92	0.45	1.91 ± 1.21	1.82 ± 1.20	0.64
Vitamin E (mg/day)	17.54 ± 8.72	20.14 ± 12.16	0.21	15.06 ± 7.77	18.04 ± 12.8	0.42	17.10 ± 9.03	16.75 ± 7.84	0.78
Alpha_ tocopherol (mg/day)	12.32 ± 5.15	13.88 ± 10.66	0.3	12.01 ± 5.13	12.46 ± 8.64	0.85	11.99 ± 5.8	11.45 ± 5.75	0.53
Thiamin (mg/day)	2.02 ± 0. 50	2.03 ± 0.67	0.9	2.04 ± 0.64	2.28 ± 0.80	0.36	1.99 ± 0.51	2.07 ± 0.82	0.42
Riboflavin (mg/day)	2.24 ± 0.55	2.32 ± 1.18	0.62	2.1 ± 0.57	2.34 ± 0.94	0.37	2.20 ± 0.58	2.29 ± 1.10	0.43
Niacin (mg/day)	21.28 ± 7.64	21.14 ± 7.73	0.92	21.24 ± 6.8	23.38 ± 9.87	0.48	21.05 ± 6.86	21.39 ± 8.56	0.76
Vitamin B6 (mg/day)	1.87 ± 0.80	1.92 ± 0.95	0.79	1.51 ± 0.37	1.86 ± 0.59	0.05	1.95 ± 0.54	1.93 ± 0.71	0.87
Total folate (mg/day)	596.46 ± 168.24	586.81 ± 223.14	0.8	633.77 ± 200.77	657.14 ± 367.71	0.82	610.58 ± 146.91	573.71 ± 187.49	0.13
DFE Folate (mg/day)	693.48 ± 211.27	685.88 ± 275.10	0.87	785.32 ± 296.4	765.7 ± 411.67	0.87	699.67 ± 192.54	662.47 ± 248.63	0.24
Vitamin_B12 (mg/day)	4.50 ± 2.10	4.91 ± 2.48	0.38	3.74 ± 4.95	2.36 ± 3.04	0.23	4.33 ± 2.30	4.50 ± 3.88	0.72
Biotin	32.07 ± 13.99	31.11 ± 13.83	0.73	26.42 ± 9.83	28.38 ± 8.62	0.57	31.88 ± 11.03	32.15 ± 14.37	0.88
Pantothenic (mg/day)	5.61 ± 1.72	5.48 ± 1.88	0.72	5.12 ± 1.78	5.94 ± 2.03	0.24	5.64 ± 1.47	5.65 ± 2.25	0.97
Vitamin K (mg/day)	369.0 ± 320.36	509.6 ± 2028.03	0.59	271.12 ± 236.73	332.64 ± 249.34	0.52	284.99 ± 234.96	277.35 ± 226.32	0.84
Phosphorus (mg/day)	71749.6 ± 80054.4	112468.78 ± 159732.5	0.76	71749.66 ± 80054.44	112468.78 ± 159732.57	0.51	117951.75 ± 166425.41	138691.95 ± 197671.81	0.52
Mg (mg/day)	399.40 ± 114.33	461.23 ± 204.76	0.97	399.4 ± 114.33	461.23 ± 204.76	0.29	412.49 ± 123.92	406.93 ± 130.61	0.76
Zinc (mg/day)	11.74 ± 3.97	12.44 ± 5.08	0.43	10.68 ± 3.57	13.33 ± 5.68	0.12	11.780 ± 4.24	11.82 ± 5.31	0.95
Cu (mg/day)	2.02 ± 0.55	1.93 ± 1.11	0.57	2.06 ± 0.64	2.04 ± 1.08	0.95	1.86 ± 0.67	1.73 ± 0.55	0.16
Manganese (mg/day)	5.59 ± 1.43	5.86 ± 2.97	0.51	5.35 ± 2.14	6.55 ± 2.48	0.16	5.86 ± 1.89	5.45 ± 2.02	0.16
Selenium (mg/day)	90.3 ± 24.89	101.16 ± 36.6	0.7	91.59 ± 32.72	100.53 ± 38.15	0.49	101.43 ± 35.06	94.35 ± 38.69	0.19
Fluorine (mg/day)	2570.5 ± 1604.7	2522.34 ± 1709.09	0.89	2697.38 ± 1783.86	2880.0 ± 2183.65	0.81	2769.08 ± 1945.10	2581.65 ± 1904.28	0.55
Chrome (mcg/da)	0.04 ± 0.04	0.07 ± 0.07	0.06	0.06 ± 0.75	0.08 ± .073	0.62	0.079 ± 0.81	0.07 ± 0.82	0.86
Total Fibre (g/day)	29.56 ± 13.99	31.78 ± 20.48	0.5	28.68 ± 12.62	31.18 ± 31.38	0.76	33.20 ± 12.96	30.61 ± 14.91	0.21
Soluble fibre (g/day)	2.18 ± 1.46	1.71 ± 1.49	0.18	1.41 ± 1.06	2.05 ± 2.06	0.34	1.73 ± 1.44	1.80 ± 1.44	0.77
Insoluble Fibre (g/day)	578.96 ± 680.15	650.65 ± 1342.22	0.77	369.05 ± 429.55	369.26 ± 847.17	0.99	498.58 ± 621.79	522.89 ± 762.7	0.84
Crude Fibre (g/day)	37.55 ± 31.01	26.84 ± 24.66	0.13	41.84 ± 33.38	38.89 ± 26.95	0.8	36.28 ± 42.52	34.79 ± 32.81	0.83
Sugar (g/day)	142.44 ± 52.36	148.23 ± 58.26	0.6	125.51 ± 45.84	152.55 ± 47.22	0.12	150.16 ± 50.85	151.84 ± 55.67	0.83
Glucose (g/day)	20.16 ± 14.20	19.96 ± 10.09	0.94	16.71 ± 13.56	21.86 ± 10.97	0.29	22.46 ± 12.26	21.06 ± 11.19	0.43
Galactose (g/day)	4.84 ± 2.58	4.84 ± 3.01	0.24	3.22 ± 3.11	5.70 ± 4.14	0.07	4.21 ± 2.91	5.52 ± 8.01	0.12
Fructose (g/day)	24.91 ± 15.4	23.23 ± 12.09	0.59	17.28 ± 16.59	22.31 ± 14.7	0.41	26.80 ± 13.55	24.01 ± 12.07	0.16
Sucrose (g/day)	48.49 ± 37.33	59.82 ± 36.95	0.15	69.48 ± 40.37	58.54 ± 38.35	0.47	54.64 ± 35.93	55.4 ± 33.22	0.88
Lactose (g/day)	16.957 ± 9.02	15.62 ± 10.24	0.51	15.11 ± 11.01	18.01 ± 12.62	0.51	15.13 ± 9.18	17.8 ± 21.24	0.23
Maltose (g/day)	2.06 ± 1.09	2.25 ± 1.39	0.44	2.52 ± 1.70	2.08 ± 1.43	0.45	2.04 ± 1.0	2.24 ± 1.46	0.27
Caffeine (mg/day)	120.53 ± 135.66	138.23 ± 102.75	0.77	64.97 ± 69.28	139.61 ± 176.12	0.39	123.85 ± 95.18	114.43 ± 115.69	0.73

**Table 4.** Linear regression of the association between the number of spontaneous abortions and dietary carotenoids

	Beta carotene		Alpha carotene		Lutein		Beta cryptoxanthin		Lycopene	
	Beta	P	Beta	P	Beta	P	Beta	P	Beta	P
Model 1	−0.04	0.73	−0.27	0.57	−0.003	0.98	0.87	0.10	−0.39	0.43
Model 2	−0.05	0.67	−0.03	0.53	0.005	0.97	0.08	0.11	−0.03	0.49
Model 3	−0.04	0.73	−0.02	0.60	−0.003	0.98	0.08	0.11	−0.04	0.39
Model 4	−0.04	0.72	−0.02	0.61	−0.002	0.99	0.08	0.12	−0.04	0.39

Model 1: Adjusted for other carotenoids, Model 2: further adjusted for age, Model 3: further adjustment for BMI and physical activity, smoking, and alcohol drinking, Model 4: further adjusted for calorie intake.

As shown in Table 5, there was a significant inverse association between dietary intake of beta-cryptoxanthin and SA in carriers of TT genotype of *FTO rs9939609* polymorphism (Beta: −0.25, *P* = 0.02) after adjustment for other carotenoids (Model 1). The relationship remained significant after further adjusting for age (Beta: −0.25, *P* = 0.02) (Model 2), additional adjusting for BMI and physical activity, smoking, and alcohol drinking (Beta: −0.26, *P* = 0.03) (Model 3), and further adjustment for calorie intake (Beta: −0.28, *P* = 0.02) (Model 4). No significant association was between dietary intake of beta-carotene, alpha-carotene, lutein, and lycopene with abortion in different *FTO* genotypes.

**Table 5.** Linear regression of the association of the number of spontaneous abortions and the intake of different types of dietary carotenoids considering *FTO* genotypes

		Model 1		Model 2		Model 3		Model 4	
		Beta	P	Beta	P	Beta	P	Beta	P
TT	Beta carotene	−0.22	0.55	−0.28	0.46	−0.23	0.56	−0.12	0.76
	Alpha carotene	−0.03	0.69	−0.05	0.60	−0.03	0.70	−0.04	0.66
	Lutein	0.060	0.86	0.11	0.76	0.06	0.87	−0.02	0.94
	Beta cryptoxanthin	−0.25	0.02	−0.25	0.02	−0.26	0.03	−0.28	0.02
	Lycopene	−0.14	0.14	−0.14	0.13	−0.14	0.15	−0.14	0.13
AA	Beta carotene	0.45	0.08	0.45	0.11	0.47	0.11	0.400	0.16
	Alpha carotene	−0.15	0.49	−0.15	0.50	−0.03	0.88	−0.039	0.86
	Lutein	−0.27	0.25	−0.27	0.26	−0.42	0.10	−0.339	0.16
	Beta cryptoxanthin	−0.05	0.77	−0.05	0.78	−0.12	0.54	−0.236	0.25
	Lycopene	−0.15	0.42	−0.15	0.43	−0.39	0.11	−0.492	0.04
AT	Beta carotene	−0.09	0.26	−0.10	0.25	−0.10	0.26	−0.10	0.23
	Alpha carotene	−0.01	0.84	−0.01	0.83	−0.009	0.91	−0.005	0.95
	Lutein	0.02	0.75	0.02	0.74	0.03	0.71	0.02	0.76
	Beta cryptoxanthin	0.05	0.49	0.04	0.53	0.05	0.47	0.05	0.51
	Lycopene	0.00	0.95	0.009	0.90	0.006	0.94	0.004	0.96

Model 1: Adjusted for other carotenoids, Model 2: further adjusted for age, Model 3: further adjustment for BMI and physical activity, smoking, and alcohol drinking, Model 4: further adjusted for calorie intake.

## Discussion

**Fig. 1.**
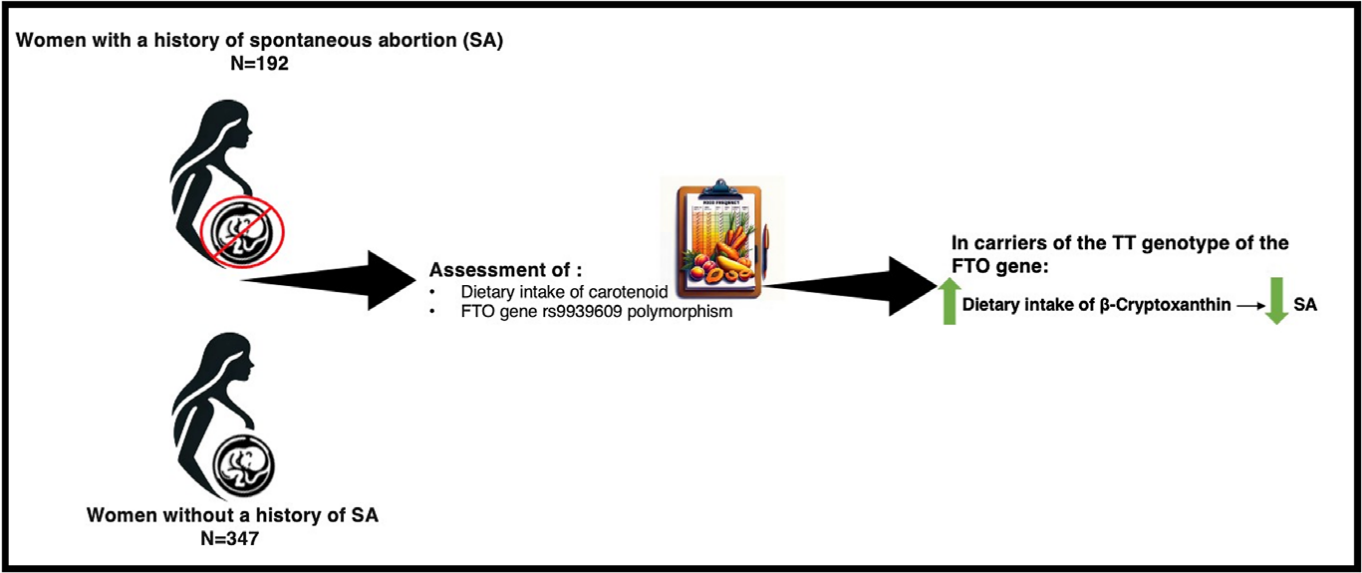
The association between dietary intake of beta-cryptoxanthin and spontaneous abortion (SA) in carriers of the TT genotype of the *FTO rs9939609* polymorphism.

The aim of the present study was to investigate the interactions between the dietary intake of carotenoids, the *FTO* gene, and SA. The results indicated a significant inverse association between dietary intake of beta-cryptoxanthin and abortion in carriers of the TT genotype of the *FTO rs9939609* polymorphism after adjustment for age, BMI, physical activity, smoking, alcohol drinking, and calorie intake (Fig. 1). Beta-cryptoxanthin is a precursor of vitamin A, which is an essential nutrient needed for eyesight, growth, development, and immune response^(23)^. In line with the present study, recent studies indicated that vitamin A and pro-vitamin A carotenoids may have important roles in conception, implantation, placentation, and regular fetal growth^(24)^. Early pregnancy loss may be resulted from premature oxygenation of the early embryonic environment^(25)^ and a meta-analysis reported that vitamin A supplementation during pregnancy may improve hemoglobin levels and reduce anemia risk during pregnancy^(17)^.

In addition to the role of as vitamin A precursor, antioxidant property of beta-cryptoxanthin is reported by several studies^(26)^. Oxidative stress has been reported to be implicated in the risk of SA^(16,27)^. Oxidative stress in the placenta and synthetic trophoblast cells was reported to induce SA^(28)^. Moreover, lower level of superoxide dismutase (SOD) as an antioxidant enzyme was detected in the homologous villi tissue of the SA group compared to the controls^(29)^. Another study reported that circulating levels of the oxidative stress biomarker (malondialdehyde) were significantly higher in individuals with recurrent SA as compared to the control group (*P*<0.05)^(30)^. Furthermore, regarding the association of dietary antioxidants with abortion, the result of several studies found a statistical difference in the serum level of some other antioxidants such as zinc^(11,31,32)^, copper, and selenium^(11,31)^ in women who had a history of recurring abortions in comparison to the control group.

In terms of the effect of *FTO* genotype on the risk of abortion, Qiu et al. found that the RNA demethylase *FTO* was downregulated in the chorionic villi of women that underwent SA, and was correlated with oxidative stress and aberrant m6A accumulation at the maternal-fetal interface^(8)^. Moreover, the role of *FTO* gene in oxidative processes and the function of antioxidants in the body was reported in previous studies^(20,33,34)^. Also, *FTO* gene is an important RNA methylation modulator gene and increased m6A RNA modification is related to the inhibition of the Nrf2-mediated antioxidant response^(35)^. Interestingly, some studies indicated that the rs9939609 polymorphism in the *FTO* gene may influence the effects of carotenoids in the human body^(36)^. So, the imbalance between the body’s antioxidant defense system and the state of free radical production can be considered as a possible mechanism of the effects of the FTO gene and dietary carotenoids on SA. However, the exact mechanisms underlying this association remain unclear and require further investigation.

The present study is the first study that identified that carotenoids (specifically beta cryptoxanthin) may play a protective role against SA only in people without the *FTO* gene allele risk of *rs9939609* polymorphism. However, the present study had some limitations. A notable limitation to be mindful of in this study is that, as a hospital-based case–control investigation on abortion, it specifically involves women who undergo SA and necessitate hospital admission. This, however, results in the exclusion of women experiencing subclinical abortions or very early pregnancy losses. Also, information about food intake was collected using self-report tool and there is a possibility of under-reporting and over-reporting. Furthermore, while this study evaluated various types of carotenoids, it did not assess overall dietary patterns or other dietary confounding factors that could influence the relationship between carotenoid intake and SA. In addition, while the study genotyped the FTO gene for the rs9939609 polymorphism, it did not consider other potential genetic factors that may interact with carotenoid intake and influence SA risk. Moreover, this study was conducted in a specific Iranian population, and the findings may not be generalizable to other populations with different dietary habits, genetic backgrounds, and environmental exposures. While the study found an inverse association between beta-cryptoxanthin intake and SA in carriers of the TT genotype of the FTO gene, the mechanisms underlying this association remain unclear. Future studies should employ larger sample sizes, utilize more objective measures of dietary intake, consider potential confounding variables, and explore additional genetic factors to better understand the complex relationship between dietary factors, genetics, and SA risk.

### Conclusion

In conclusion, the current study demonstrated that a lower intake of cryptoxanthin, coupled with specific *FTO* genotypes (specifically the TT genotype), may increase the risk of SA. Further studies and meta-analyses are necessary to determine the role of carotenoids in the risk of SA in people with different *FTO* genotypes. Further research is required to confirm the presumed connection between the *FTO* gene, dietary carotenoids, and the risk of SA and to discover the underlying mechanisms. If the results of this study are confirmed, the consumption of dietary carotenoids or supplements containing carotenoids can be recommended in people who are genetically prone to abortion.

## Data Availability

The datasets used and/or examined in the present investigation may be obtained from the corresponding author upon reasonable request.
